# Serial dependence and representational momentum in single-trial perceptual decisions

**DOI:** 10.1038/s41598-021-89432-9

**Published:** 2021-05-10

**Authors:** D. Pascucci, G. Plomp

**Affiliations:** 1 Department of Psychology, University of Fribourg, Fribourg, Switzerland; 2 Laboratory of Psychophysics, Brain Mind Institute, École Polytechnique Fédérale de Lausanne (EPFL), SV 2805 (Bâtiment SV) Station 19, 1015 Lausanne, Switzerland

**Keywords:** Decision, Perception

## Abstract

The human brain has evolved to predict and anticipate environmental events from their temporal dynamics. Predictions can bias perception toward the recent past, particularly when the environment contains no foreseeable changes, but can also push perception toward future states of sensory input, like when anticipating the trajectory of moving objects. Here, we show that perceptual decisions are simultaneously influenced by both past and future states of sensory signals. Using an orientation adjustment task, we demonstrate that single-trial errors are displaced toward previous features of behaviorally relevant stimuli and, at the same time, toward future states of dynamic sensory signals. These opposing tendencies, consistent with decisional serial dependence and representational momentum, involve different types of processing: serial dependence occurs beyond objecthood whereas representational momentum requires the representation of a single object with coherent dynamics in time and space. The coexistence of these two phenomena supports the independent binding of stimuli and decisions over time.

## Introduction

Our visual world exhibits strong temporal dependencies: previous events largely determine events in the present and future. This allows our brain to build expectations about its visual input based on prior experience^1,2^. For example, we can expect that features of a static scene remain constant over time, or that objects in motion continue to move along their paths and trajectories. These expectations ultimately prime our perceptual system toward the stability or change of events in the environment, leading to systematic biases in our perceptual representations and decisions.

Two distinct types of biases, known as serial dependence (SD)^3^ and representational momentum (RM)^4^, have been linked to expectations about stable or dynamic sensory signals. In SD, small and unpredictable changes in otherwise stable visual input are underestimated and we perceive present stimuli as more similar to the past than they are. In RM, perceptual reports are instead biased toward future states along the predictable trajectory of dynamic stimuli. These opposite phenomena suggest that perceptual representations tend to either stabilize or to be displaced forward, depending on the expected dynamics of sensory events.

For instance, in a typical SD paradigm involving orientation adjustment responses, participants reproduce a feature (e.g., the orientation of a Gabor patch) in a series of trials. Adjustment responses are found to be systematically biased toward orientations seen one or a few trials before, even though stimuli are presented in a random sequence and with relatively long inter-trial intervals (e.g., 5–10 s)^3^. SD occurs mostly for weak (e.g., low contrast) and briefly presented sensory signals, and shows a broad spatial tuning (e.g., ~ 15^3^). Recent work suggests that SD may originate beyond early sensory processing (e.g., during decision or memory processes^5,6^) and acts directly on the perception of the next stimulus^7^. This phenomenon has been related to predictive processes by which an internal representation of the previous stimulus is used as a prior for current perception^7,8^.

In a typical RM experiment, participants are exposed to a sequence of stimuli that contain regular dynamics (e.g., rotating rectangles). Contrary to SD, perceptual judgments and recognition memories for the last stimulus tend to be displaced forward, in the direction of the implied dynamic (e.g., an oriented rectangle is reported as more clockwise than it is, after a sequence of rectangles rotating clockwise^4^). The forward displacement depends on the coherent path of the preceding stimuli and disappears when the sequence is shuffled, and the implied dynamic disrupted^4,9,10^. RM is affected by a variety of factors, including dynamic properties of stimulus such as velocity and acceleration^9^ and higher-level aspects, such as expectations and semantic knowledge (e.g., a stimulus named “rocket” induces larger RM than a stimulus named “cathedral”^11^). RM also requires that the identity of the stimuli remains constant during the sequence, as in a single and coherently changing object^12^. It is generally believed that RM reflects high-level and memory-related processes, rather than low-level sensory biases^10^, which may rely on internalized physics of sensory events to bridge the ‘gap’ between perception and action^9^.

Hence, both SD and RM have been linked to expectations and predictive processes that may be triggered, respectively, by the unpredictability or coherent behavior of a sequence of stimuli. In the present work, we show that SD and RM can be also present at the same time. Despite their simultaneous presence, however, we demonstrate that they rely on different types of processing. With the present paradigm, we found positive SD exclusively for sequential decisions, independently of whether stimuli were perceived as coherent objects. RM required the representation of a single object with coherent spatiotemporal dynamics^9,13,14^.

## Results

**Figure 1:**
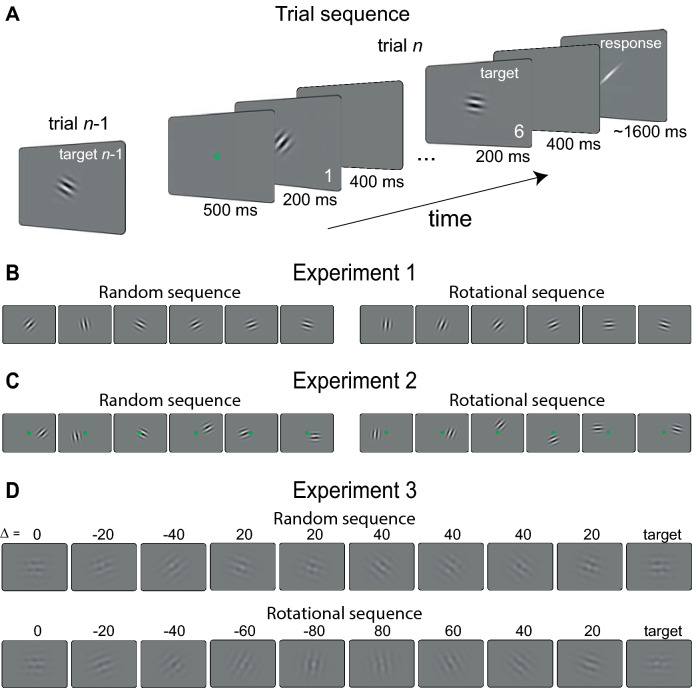
(**A**) Example of a trial sequence. On each trial, participants were presented with a sequence of oriented Gabors and reproduced the orientation of the last one by adjusting a response bar. (**B**) The two conditions of Experiment 1, with stimuli presented at fovea. In separate blocks of trials, the orientation sequence unfolded either randomly or following a rotational momentum (here a clockwise rotation). (**C**) In Experiment 2, stimuli were presented at random locations within ± 3° off fixation. (**D**) In Experiment 3, longer sequences (from 4 to 12) of low-contrast and noisy Gabor stimuli were used. The Random sequences were designed to yield stronger adaptational bias than the Rotational condition (e.g., more Gabors tilted in the same direction relative to the target, here clockwise, Δ = 20°–40°). The green fixation spot was presented at the beginning of each sequence in Experiment 1 and 3 and remained on screen for the entire sequence in Experiment 2. Stimuli are not drawn to scale.

**Figure 2:**
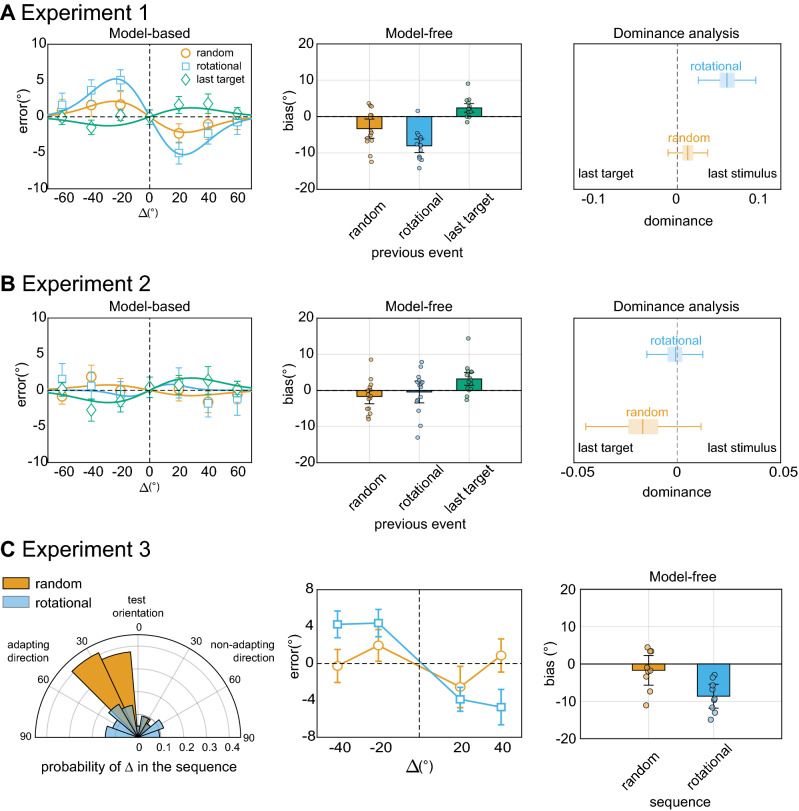
(**A**) Results for model-based, model-free, and dominance analyses of Experiment 1. Repulsive adaptation followed random sequences of sensory signals (orange circles, curves, and bar). The presence of rotational structures pushed perceptual reports further away from previous stimuli and toward future states (sky blue circles, curve, and bar). Previously reported orientations (last target) had the opposite effect, inducing serial dependence toward the past (green diamonds, curve, and bar). The bias induced by rotational sequences dominated perceptual reports (boxplots on the right side). (**B**) Results of Experiment 2. Presenting stimuli at different retinal locations removed both adaptation and RM effects, while SD by previous targets remained significant. In this experiment, the bias toward the past dominated, particularly during random sequences of stimuli (boxplots on the right side). (**C**) Results of Experiment 3. The polar plot on the left shows the proportion of adaptors shown in this experiment, to induce more adaptation bias in the Random compared to the Rotational condition. The central plot shows the average error over Δ in the two conditions (mean-corrected per condition), revealing larger repulsive biases for rotational than for random sequences, despite the designed asymmetry in adaptation bias. The use of noisy and low-contrast Gabors removed overall adaptation effects whereas momentum effects remained significant (model-free results, bar plot on the right side). Error bars are standard errors of the mean in line plots and 95% CI of the mean in bar plots.

In Experiment 1, fourteen participants were presented with a sequence of six low-contrast Gabor stimuli and were asked to reproduce the orientation of the last one using adjustment responses (Fig. 1A). Stimuli were presented at the same foveal location and the sequence of orientations unfolded either randomly (Random) or followed a regular rotational trajectory (Rotational) (Fig. 1B). We compared response errors in the two conditions to test whether perceptual biases would exhibit RM in Rotational trials and SD in Random trials. To this aim, we performed model-based and model-free analyses on the adjustment errors (reported minus actual orientation) as a function of the orientation difference between the target stimulus (the last one in the sequence) and the preceding one (Δ, previous minus present orientation, see “Methods” section). The Δ between the last two stimuli was equally varied (from ± 20° to ± 60°) across conditions, but the preceding history changed, containing either rotational sequences or randomly shuffled sequences. Following the convention used in previous work^3,5^, errors and Δ with the same sign indicated a bias in adjustment responses toward the previous orientation (i.e., attractive, positive sign). Errors and Δ with opposite sign indicated a bias away from the previous orientation (i.e., repulsive, negative sign) (see “Methods” section and Fig. 2). In this convention, which is different from the one typically used in the RM field (see “Methods” section), SD is consistent with an attractive bias^3^ whereas RM is consistent with a repulsive bias: an overestimation of the difference between the present and the previous orientation. These terms are synonyms of the backward and forward displacement in the RM literature and the error measure is equivalent to the standard *M*-displacement measure in RM studies but with the inverted sign^9^.

We found no significant attractive SD biases after Random sequences, but a robust repulsive effect of previous stimuli, with a peak negative bias of 2.16° at Δ =  ± 24°, corresponding to a deviation of about 8% toward the previous orientation (see “Methods” section; model fitting analysis: bootstrap *p* < 0.001; model-free analysis: *t*(13) = − 2.44, *p* = 0.02, two-tailed t-test against zero, Cohen’s *d* = 0.65; Fig. 2A; see “Methods” section). The presence of explicit rotational dynamics further increased the repulsive bias by a factor of almost three times (peak negative bias of 5.26° at Δ =  ± 23°, deviation of 22%; model fitting analysis: bootstrap *p* < 0.001; model-free analysis: *t*(13) = − 7.53, *p* < 0.001, Cohen’s *d* = 2.01), producing a statistically larger bias in the Rotation than the Random condition (model fitting analysis: bootstrap *p* < 0.001; model-free results: *t*(13) = 4.25, *p* < 0.001, paired t-test, Cohen’s *d* = 1.02; see “Methods” section).

In Experiment 1, the repulsive bias after random sequences closely resembled well-known adaptation aftereffects in which perception is shifted away from features exposed in the past, a phenomenon that originates at the early stages of visual processing^5,15,16^. When rotational structures were embedded in the sequence, RM added on neuronal adaptation, biasing perceptual reports further away from the past and toward the future.

The absence of SD in random sequences of non-reported stimuli replicates previous findings using a similar paradigm^5^. However, this result is not unequivocal. There are at least two alternative explanations. Following previous work, one possibility is that SD only emerges for stimuli that are relevant for behavior and undergo complete attentional and decision-making processing^5,6,17^. Alternatively, SD may have been hindered by adaptation, since the two phenomena are likely to co-exist and interact in perceptual processing^5^. To evaluate the first possibility, we measured residual biases in adjustment responses due to the orientation reported in the previous trial. This revealed a positive bias of about 4% toward the target orientation reported in the last trial (model fitting analysis: bootstrap *p* < 0.001; model-free analysis: *t*(13) = 3.39, *p* = 0.004, Cohen’s *d* = 0.90), with a peak of 1.24° when the difference between consecutive orientations (Δ) was 28.3°, in line with the shape and size of SD reported before^5,6^.

In a second experiment (Experiment 2, fifteen participants) we tested whether SD would emerge also for non-reported stimuli in the absence of adaptation. This remains a fundamental aspect to validate previous work and conclusions on the nature of SD^5^. Adaptation to oriented stimuli involves sensitivity changes in early visual neurons that are selective for retinal locations, whereas SD has a more broad retinotopic organization^18,19^. Thus, if the two phenomena interfere with each other, sequential stimuli at different locations should prevent adaptation and release serial dependency effects. To address this possibility, we used the same sequences and conditions as in Experiment 1, but stimuli were presented at different, randomly assigned locations (within ± 3° off fixation; Fig. 1C). By preventing repeated stimulation at the same retinal location, repulsive effects disappeared completely (Fig. 2B; model fitting analysis: both Random and Rotational, bootstrap *p* > 0.05; model-free analysis: both *p* > 0.05, all Cohen’s *d* < 0.40). However, SD by reported orientations was unaffected (peak bias of 1.70° at Δ =  ± 28°, deviation of about 6% toward the previous orientation, model fitting analysis: bootstrap *p* < 0.001; model-free analysis: *t*(14) = 3.02, *p* = 0.009, Cohen’s *d* = 0.78), despite the target stimulus in the last trial and the present one could occur six degrees away in retinal coordinates.

Our first two experiments confirmed that RM requires the representation of a single visual object that exhibits coherent dynamics in space and time^4,9^. The simultaneous presence of adaptation and RM, however, partly confounded the true nature of this effect. For instance, the observed displacement of errors in the direction of rotation could simply result from stronger adaptation effects induced by sequences containing more orientation signals in the same direction. For instance, a Δ of − 20° is always preceded by a Δ of − 40° in a rotational but not in a random sequence. To disambiguate between low-level adaptation and RM, we performed a final experiment designed to (1) reduce overall adaptation effects and (2) systematically present more adapting orientations in the Random than in the Rotational condition (see “Methods” section and Figs. 1D, 2C). If the findings of Experiment 1 were exclusively due to adaptation, these manipulations should result in larger repulsive biases in the Random condition, due to the larger number of adapting orientations (Fig. 2C). At the same time, biases due to implied rotation were less likely to occur in Random sequences, where a rotational component was prevented. The results in nine participants revealed RM effects beyond any effect of adaptation. Despite the increased number of adaptors in the Random condition, repulsive biases were larger for rotational than for random sequences (model-free analysis: *t*(8) = − 4.67, *p* = 0.002, paired *t* test; Cohen’s *d’* = 1.56). Furthermore, for the low-contrast and noisy Gabors used here, pure adaptation aftereffects were not significant (model-free analysis: *t*(8) = − 0.98, *p* = 0.35, paired t-test). Yet, adjustment errors in the Rotational condition deviated significantly from zero (model-free analysis: *t*(8) = − 6.20, *p* < 0.001, paired *t* test, Cohen’s *d* = 2.06).

Collectively, our experiments showed distinct biases arising from the history of sensory processes, internalized dynamics, and behaviorally relevant sensory signals. This further established the existence of opposing forces in single-trial perceptual reports, causing either repulsion and RM or SD^5^. To evaluate the relative impact of these opposing forces, in a final step we performed a dominance analysis (see “Methods” section) comparing the effect of non-reported and reported orientations in the first two experiments. The analysis revealed a dominance of recent stimuli over previously reported ones in Experiment 1, where sensory stimulation was fixed at the fovea, with rotational sequences exerting the largest effect on perceptual biases (Fig. 2A,B, rightmost panels). Conversely, positive dependencies by reported orientations dominated when stimuli occurred at different spatial locations, although their dominance was less pronounced in Rotational trials.

## Discussion

Overall, our results support two main conclusions. First, temporal structures in sensory input produce robust biases in perceptual reports toward the future state of stimuli, confirming a large body of work on RM. RM dominates over concurrent history effects and requires the representation of a single and coherent visual object. Second, SD does not retain the same degree of objecthood and is promoted by task-relevance and active behavior. The absence of RM in Experiment 2, despite explicitly cueing participants about the regularity or randomness before each block (see “Methods” section), indicates that the effect depends not only on the rotation per se but also on the ecological validity of stimulus (e.g., a single spinning object is unlikely to change position randomly in the short term).

Previous work has documented several factors operating in the direction opposite to RM, like central tendency effects^20^ and compensation biases^21^. These effects are typically due to processes that emerge with longer retention intervals, such as memory shifts toward the average state of a sequence of stimuli^20^. Here we show that SD biases, also operating in the direction opposite to RM, can be observed simultaneously to RM.

Our findings have important implications for the current perspectives of SD. The first is that SD depends mostly on behaviorally relevant stimuli, even when adaptation aftereffects are controlled for. This challenges the general idea that perception is fundamentally biased toward prior sensory events, a tenet of Bayesian models of SD^22–24^. Here we show that the simple exposure to sequences of random orientations, although attended and consciously perceived, produces no systematic bias at all. This was observed even after controlling for adaptation confounds and at a time scale where previous stimuli are believed to reflect informative priors in more naturalistic contexts (i.e., less than 4 s, the duration of our sequence)^24^. On the contrary, a robust perceptual bias was observed at the same time scale following rotational sequences, consistent with the effect of expectations and anticipatory mechanisms, largely involved in RM^11,25^. An alternative possibility is that SD for stimuli within the trial sequence may have been reduced by the short inter-stimulus interval (400 ms, compared to seconds in typical SD paradigms). This possibility, however, would confirm that SD and RM rely on different types of processing and different time scales. The second clear implication is that attractive and repulsive biases engage different stages of processing and may not rely on the same history. While it is assumed that SD reflects biased decoding of sensory information that dominates over adaptation for more recent trials^22^, our results demonstrate that the two phenomena track a different and dissociable history of events: in our paradigm, adaptation depended on sequences of sensory events; SD depended on sequences of perceptual decisions and was therefore independent of the pure history of stimulation. This supports a recent hierarchical view of history biases^5^ and places evident constraints on theoretical and computational models of history biases. The observed dissociation between SD and sensory history, for instance, is not entirely consistent with a pure Bayesian observer model where recent sensory input systematically acts as prior for present perceptual decisions^22^.

Our results demonstrated that SD emerges at later stages than early sensory cortices^7^, likely involving task-related processing^5^. We cannot rule out the existence of SD at multiple stages of processing, as hypothesized in previous work^26^. From our results, perceptual decisions appeared necessary for SD, but decisional processes alone may not be sufficient and the interplay between decisions and the specific type of perceptual processing required by a task may play a role as well. Here we showed that for stimuli requiring identical processing (e.g., orientation signals), positive SD was promoted by past decisions rather than by the mere history of stimuli. In line with this result, recent work has shown that SD is reduced during changes in secondary features (e.g., changes in stimulus color during motion direction judgments) but only when those features are relevant for the current task^27^. This suggests that SD emerges from task processing, rather than from the pure exposure to sensory signals. Note that this conclusion refers to the *source* of SD. Our study does not exclude that SD ultimately leads to changes in the appearance of stimuli, i.e., that the *source* is post-perceptual but the *site of action* is perceptual^7^. Perceptual decisions^5^ and higher-level cognitive biases may still propagate down to sensory cortices, directly distorting the appearance of stimuli^7^.

A third important aspect of our results is that SD and RM operate on different representations. While RM seems to rely on predictions about the coherent spatiotemporal dynamics of a single object, SD occurs at a more abstract level of representation, where the only dimension of sensory input that counts is the one that is relevant for decisions (e.g., orientation but not spatial location). This does not exclude, however, the involvement of predictive processes in SD. Expectations may indeed be formed but at the level of sequential decisions in which only the task-relevant dimension of sensory input acts as a prior. The requirement of a task may bind together decisions and relevant features of stimuli into an *event file* that is expected to be reactivated soon^28^, weighting more their related representations than the history of unreported features and stimuli. In this view, SD in perceptual biases is a sequential effect of decisions on a specific dimension of sensory input, which may be independent of all other aspects of stimuli^17^. Interestingly, this would even predict SD for completely different stimuli, provided that they share a common task-relevant dimension^29^.

In sum, we show that single perceptual reports contain signatures of both future and past states of sensory signals. This conclusion holds under the paradigm employed here, where the history of stimuli and the history of decisions were independently manipulated. Nevertheless, the results provide evidence against the general notion that perception is fundamentally biased towards past states. The two opposite biases reported here, originated from different types of processing, where perception and decisions follow the independent history of stimuli and task-relevant behavior.

## Methods

### Ethics statement

The study was approved by the local ethics committee of the University of Fribourg and of the École Polytechnique Fédérale de Lausanne (EPFL) and carried out under the Declaration of Helsinki.

### Participants

A total of forty healthy human subjects from the University of Fribourg and EPFL participated in the study for course credits or monetary reward (30 CHF). Fifteen subjects participated in Experiment 1 (13 females, mean age of 20.26 ± 1.03 years) and in Experiment 2 (10 females, mean age of 21.66 ± 2.49 years). Ten subjects participated in Experiment 3 (3 females, mean age of 23 ± 3.82). All participants had normal or corrected-to-normal vision and were naïve as to the purpose of the experiments. Written informed consent was collected from all participants in advance.

### Apparatus

Stimuli were presented on a Multiscan G500 CRT 21″ monitor (1024 × 768 pixels, 100 Hz; Experiment 1–2) and on a gamma-corrected VG248QE monitor (1920 × 1080 pixels, 120 Hz, Experiment 3) and were generated with custom-made scripts written in Matlab and the Psychophysics Toolbox^30^, running on Windows-based machines. Experiments were performed in a darkened room and participants sat at 70 cm (Experiment 1–2) and 57 cm (Experiment 3) from the computer screen, with their head positioned on a chinrest. All stimuli were presented on a grey background.

### Stimuli and procedures

An example of a trial sequence in Experiment 1 is illustrated in Fig. 1A. Each trial started with a central green fixation spot (0.5°, 500 ms) followed by the presentation of a sequence of Gabor stimuli at the fovea. Gabor stimuli had a peak Michelson contrast of 25%, spatial frequency of 1.2 cycles per degree, and a Gaussian contrast envelope of 1.5°. In 80% of trials, the sequence was composed of six Gabor stimuli (200 ms each) interleaved with 400 ms of blank interval^5^. On the remaining 20% control trials, the sequence ended at a random position before the sixth Gabor (after 1–5 Gabors). Control trials were included to control that participants were paying attention to the entire sequence and, most importantly, to the fifth stimulus before the to-be-reported orientation (fifth versus sixth stimulus error magnitude, Experiment 1: *t*(13) = 0.009, *p* = 0.99; Experiment 2: *t*(14) = 1.86, *p* = 0.08, two-tailed *t *test). The task required participants to reproduce the orientation of the last-seen Gabor by adjusting a response bar with the computer mouse. We also included 16% of additional control trials in the Rotational condition in which the last orientation was chosen randomly, not following the expected sequence. These trials were included to address whether, in addition to RM, predictable sequences would also increase the precision of behavioral reports. The magnitude of errors in these trials, however, was not significantly different from trials with complete rotational sequences (Experiment 1: *t*(13) = 1.28, *p* = 0.22; Experiment 2: *t*(14) = − 0.28, *p* = 0.77, two-tailed *t *test).

For each sequence, the orientation of the first stimulus was randomly selected in the 0°–160° range (steps of 20°). In each trial of the Rotational condition, changes in the orientation of consecutive stimuli were created by randomly selecting a delta orientation (the difference between previous and present orientations, Δ) within the ± 60° range (in steps of 20°), and keeping the Δ fixed within one trial, to mimic a constant rotational motion in either the clockwise or counter-clockwise direction. Trials of the Random condition were created by shuffling the order of stimuli in the Rotational condition with the constraint to maintain the same number of data points for each Δ but removing any temporal structure from the sequence. No noise mask was presented after each stimulus, and the phase of the sinusoidal component of each Gabor was changed randomly on each presentation (0°–360°, steps of 15°) to minimize contrast aftereffects. After 400 ms from the offset of the last Gabor in the sequence, the response bar appeared at the center of the screen, and participants had to report the orientation of the last stimulus. Only trials in which the sequence was completed after six Gabors were analyzed. There were four blocks of 72 trials each, for a total of 288 trials. One subject in Experiment1 collected only 192 trials due to computer failure. Stimuli and procedures were identical in Experiment 2, with the exception that Gabor stimuli were presented at random locations within a ± 3° radius from the monitor center, and the green fixation spot remained on screen.

In Experiment 3, the stimuli and the sequence were modified to control for the effect of adaptation. To reduce the adapting strength of each stimulus, Gabors were linearly combined with Gaussian-windowed white-noise patches, filtered at the same spatial frequency of the Gabor. The resulting stimulus (50% of Gabor and 50% of noise, see Fig. 1D) was presented with low contrast (peak Michelson contrast of 10%). Random sequences were designed to contain more potential adaptors than rotational sequences (e.g., more orientation signals in the same ± 20, 40° direction to the target). More precisely, in both conditions, we used longer sequences, from 4 to 12 stimuli. In the Rotational condition, the difference between consecutive orientations was fixed at either ± 20° or ± 40°. In this way, a rotational sequence could cover the whole orientation range but included at most two Δ, the second-and third-to-last, that can exert a pure adaptation-like bias (i.e., Δ of ± 20° or 40°, as observed in Experiment 1). In the Random condition, stimuli with Δ larger than ± 40° were replaced by stimuli having Δ in the adapting range (e.g., ± 20 and ± 40° relative to the target). Thus, we obtained sequences where the rotational component was disrupted but the overall number of effective adaptors was increased (see Fig. 2C). There were four blocks of 80 trials each, for a total of 320 trials. All other aspects were the same as in Experiment 1.

In all experiments, at the beginning of each block, participants were informed by a written cue about the regularity or randomness of the upcoming block. Participants performed a brief practice session (~ 20 trials) before each experiment.

### Analysis

Before statistical analysis, trials containing absolute adjustment errors larger than 3 standard deviations from the participant’s mean and reaction times smaller than 500 ms or larger than 10 s were marked as outliers (less than 5% in total). Control trials, outlier trials, and trials immediately after were removed from subsequent analyses. Adjustment errors were computed as the mean-corrected acute angle of the difference between reported and actual orientations. Participants performed the adjustment task with an average absolute error of 9.24 ± 1.68° in Experiment 1,10.86 ± 2.96° in Experiment 2 and 11.52 ± 4.70 in Experiment 3. The average reaction times were 1.56 ± 0.27 s in Experiment 1, 1.67 ± 0.40 s in Experiment 2, and 1.51 ± 0.34 in Experiment 3. One participant from Experiment 1 and one from Experiment 3 was excluded from the analysis because of a standard deviation in adjustment errors larger than 30°.

It should be noted that the analysis and related terminology employed in this study follow standards in the field of SD^3,5,31^. This convention is sometimes different from the one used in RM works. For instance, we used the term “attractive” or “repulsive” to indicate biases in adjustment responses toward or away from the previous stimulus orientation. Moreover, in some analyses (e.g., the model-free analysis, see below), we represent them using positive and negative signs, respectively. Typically, similar biases are referred to as backward or forward displacement in RM literature and their sign is inverted^4,9,14^.

Data were analyzed both using a model-based and a model-free approach. In the model-based procedure, the strength of the adjustment bias (both negative and positive) was quantified as the amplitude parameter α of a derivative of Gaussian function (DoG)^3^:

$$error = \Delta \alpha wce^{{ - \left( {w\Delta } \right)^{2} }}$$

where $c$ is a constant $c = \sqrt 2 /e^{ - 0.5}$ and $w$ is the inverse of the curve width. The DoG amplitude parameter α quantifies the deviation of the predicted errors, in degrees, from the actual orientation, as a function of the Δ variable (the relative difference between consecutive orientations, computed as previous minus present orientation).

The amplitude of the DoG curve was estimated solving a constrained non-linear minimization problem with the sum of squared residuals as the cost function^32^. The estimation was performed at the group level, using a stratified bootstrap resampling. The resampling procedure was done by combining 80% of randomly selected trials from each participant into a grand subject dataset and reiterating 5000 times^6,33^. Model fitting was applied to each condition of interest separately. Statistical *p* values were obtained as the proportion of bootstrap α parameters below or above zero. For direct comparison between conditions, surrogate α parameters were obtained by randomly shuffling the condition labels 10,000 times and comparing the distribution of the resulting differences against the observed one. The peak bias, reported in percentage, was computed as the ratio between the maximum predicted deviation of errors from zero (e.g., the DoG peak α) and the related Δ value*.*

In the model-free approach, which served as a control analysis, we subtracted the average error for Δ of 20° and 40° from the average error in the corresponding negative Δ^31^. The resulting index, quantifying the amount of systematic deviation of the errors from zero (either in the positive or negative direction) was used for subsequent analysis. The same approach was used for the analysis of Reported Stimuli. The analysis of Experiment 3 was exclusively based on a model-free approach, given the restricted range of Δ used (± 20° and 40°).

To compare the effect of the last stimulus in the Rotational and Random conditions against the effect of the orientation reported in the previous trial, we ran a linear dominance analysis for the first two Experiments^5^. The analysis was performed using the same bootstrap procedure described above and generating two variables based on the sign of Δ for the last stimulus and the last reported orientation (Δs were restricted to ± 20°–40°, a range where the DoG approximates a linear function). The two variables were then used to predict errors in current reports. Distributions of relative predictive dominance (Δ[last stimulus] − Δ[last reported]) were obtained with the bootstrap procedure separately for the Rotational and Random conditions (Fig. 2A,B, rightmost panels).

## Data Availability

The datasets analysed in this study are available at 10.5281/zenodo.5140193
