# Author Correction: Serial dependence and representational momentum in single-trial perceptual decisions

**DOI:** 10.1038/s41598-021-96023-1

**Published:** 2021-08-12

**Authors:** D. Pascucci, G. Plomp

**Affiliations:** 1 Department of Psychology, University of Fribourg, Fribourg, Switzerland; 2 Laboratory of Psychophysics, Brain Mind Institute, École Polytechnique Fédérale de Lausanne (EPFL), SV 2805 (Bâtiment SV) Station 19, 1015 Lausanne, Switzerland

Correction to: *Scientific Reports* https://doi.org/10.1038/s41598-021-89432-9, published online 10 May 2021

The original version of this Article contained errors. The Data Availability section was not included; it has now been added and states:

**Data Availability**

The datasets analysed in this study are available at https://doi.org/10.5281/zenodo.5140193

Additionally, an error carried over from earlier version of this manuscript has been fixed in the Results, where

“However, SD by reported orientations was unaffected (peak bias of 1.86° at Δ = ± 28°, deviation of about 6% toward the previous orientation, model fitting analysis: bootstrap *p* < 0.001; model-free analysis: *t*(14) = 3.02, *p* = 0.009, Cohen’s *d* = 0.78), despite the target stimulus in the last trial and the present one could occur six degrees away in retinal coordinates.”

now reads:

“However, SD by reported orientations was unaffected (peak bias of 1.70° at Δ = ± 28°, deviation of about 6% toward the previous orientation, model fitting analysis: bootstrap *p* < 0.001; model-free analysis: *t*(14) = 3.02, *p* = 0.009, Cohen’s *d* = 0.78), despite the target stimulus in the last trial and the present one could occur six degrees away in retinal coordinates.”

The Article has now been corrected.

